# Murine models for familial pancreatic cancer: Histopathology, latency and drug sensitivity among cancers of *Palb2*, *Brca1* and *Brca2* mutant mouse strains

**DOI:** 10.1371/journal.pone.0226714

**Published:** 2019-12-26

**Authors:** Dongju Park, Reena Shakya, Christopher Koivisto, Jason R. Pitarresi, Matthias Szabolcs, Raleigh Kladney, Ashley Hadjis, Thomas A. Mace, Thomas Ludwig

**Affiliations:** 1 Department of Cancer Biology and Genetics, The Ohio State University Comprehensive Cancer Center, Columbus, Ohio, United States of America; 2 The Ohio State University Comprehensive Cancer Center, Columbus, Ohio, United States of America; 3 Institute for Cancer Genetics, Department of Pathology and Cell Biology, and Herbert Irving Comprehensive Cancer Center, Columbia University Medical Center, New York, New York, United States of America; 4 Department of Internal Medicine, The Ohio State University, Columbus, Ohio, United States of America; CNR, ITALY

## Abstract

Alterations of the *PALB2* tumor suppressor gene have been identified in familial breast, ovarian and pancreatic cancer cases. PALB2 cooperates with BRCA1/2 proteins through physical interaction in initiation of homologous recombination, in maintenance of genome integrity following DNA double-strand breaks. To determine if the role of PALB2 as a linker between BRCA1 and BRCA2 is critical for BRCA1/2-mediated tumor suppression, we generated *Palb2* mouse pancreatic cancer models and compared tumor latencies, phenotypes and drug responses with previously generated *Brca1/2* pancreatic cancer models. For development of *Palb2* pancreatic cancer, we crossed conditional *Palb2* null mouse with mice carrying the *Kras*^*G12D*^*; p53*^*R270H*^*; Pdx1-Cre* (*KPC*) constructs, and these animals were observed for pancreatic tumor development. Individual deletion of *Palb2*, *Brca1* or *Brca2* genes in pancreas *per se* using *Pdx1-Cre* was insufficient to cause tumors, but it reduced pancreata size. Concurrent expression of mutant *Kras*^*G12D*^ and *p53*^*R270H*^, with tumor suppressor inactivated strains in *Palb2*-*KPC*, *Brca1*-*KPC* or *Brca2*-*KPC*, accelerated pancreatic ductal adenocarcinoma (PDAC) development. Moreover, most *Brca1*-*KPC* and some *Palb2*-*KPC* animals developed mucinous cystic neoplasms with PDAC, while *Brca2*-*KPC* and *KPC* animals did not. 26% of *Palb2-*KPC mice developed MCNs in pancreata, which resemble closely the *Brca1* deficient tumors. However, the remaining 74% of *Palb2-KPC* animals developed PDACs without any cysts like *Brca2* deficient tumors. In addition, the number of ADM lesions and immune cells infiltrations (CD3^+^ and F/480^+^) were significantly increased in *Brca1-KPC* tumors, but not in *Brca2-KPC* tumors. Interestingly, the level of ADM lesions and infiltration of CD3^+^ or F/480^+^ cells in *Palb2-KPC* tumors were intermediate between *Brca1-KPC* and *Brca2-KPC* tumors. As expected, disruption of *Palb2* and *Brca1*/2 sensitized tumor cells to DNA damaging agents *in vitro* and *in vivo*. Altogether, *Palb2*-*KPC* PDAC exhibited features observed in both *Brca1*-*KPC* and *Brca2*-*KPC* tumors, which could be due to its role, as a linker between Brca1 and Brca2.

## Introduction

Pancreatic cancer is one of the deadliest cancer types, with a 5 year survival rate of 8%, due to the lack of early detection, which limits treatment options [1]. Despite many research efforts, initiating factors for pancreatic cancer are not well defined. An estimated 5~10% of pancreatic cancer is familial, with breast cancer susceptibility genes 1/2 (*BRCA1/2*) and partner and localizer of BRCA2 (*PALB2*) among established pancreatic susceptibility genes [2–7]. In 2016, Bailey *et al*. reported 5% germline mutations and 12% somatic mutations in the BRCA pathway (BRCA1, BRCA2, ATM and PALB2) through whole genome sequencing of 456 pancreatic cancer [8]. Therefore, it is important to understand how these genes involved in BRCA pathway contribute to pancreatic cancer development.

After discovery of *BRCA2* in 1995 [9, 10], when a homozygous deletion lying within 13q12.3 where the *BRCA2* gene resides was identified in a human pancreatic cancer [11], more germline *BRCA2* mutations were found in pancreatic cancer patients [6, 12–14]. Generation of *Brca2* pancreatic cancer mice model by pancreas specific disruption of *Brca2* gene with inactivation of *p53* determined that *BRCA2* is a bonifide pancreatic tumor suppressor gene, reflecting increased risk in *BRCA2* mutation carriers for pancreatic cancer [15–17]. Several studies reported increased cancer risk in *BRCA1* mutant carriers [5, 18, 19], although the association between BRCA1 and pancreatic cancer predisposition is not well-established [18]. Previously, we showed that Brca1 suppresses pancreatic tumor development by showing dramatically reduced tumor latency in *Brca1* deleted triple mutant animals (*Brca1*^*flox/flox*^*; Kras*^*G12D*^*; p53*^*flox7/flox7*^*;Pdx1-Cre*) [20]. More recently, the *PALB2* gene was discovered [21] when researchers were looking for genes that confer susceptibility to pancreatic cancer, and Jones *et al*. reported inherited *PALB2* mutations in familial pancreatic cancer [22]. Since then, more mutations in *PALB2* gene have been identified in pancreatic cancer [8, 23], implying the urgent need of Palb2 pancreatic cancer mouse models to understand its role in pancreatic cancer development.

PALB2 was first identified as a binding partner of BRCA2 and shown to be required for the localization of BRCA2 to sites of DNA damage, and thus crucial for homologous recombination (HR) [21]. PALB2 harbors a series of C-terminal WD repeats that bind the N-terminus of BRCA2. In addition, the coiled-coil (CC) region at the N-terminus of PALB2 interacts with the CC domain of BRCA1. Down regulation of PALB2 by siRNA suppresses HR in a manner similar to BRCA1 and BRCA2 depletion [24]. Like *BRCA1* (FANCS) [25] and *BRCA2* (FANCD1) [26], monoallelic mutations in *PALB2* confer familial susceptibility to breast, ovarian and pancreatic cancer [4, 7], while biallelic *PALB2* lesions cause Fanconi anemia (FA) subtype N (FANCN) [27]. FA patients are highly prone to cancer due to their inherited defect in FA/HR DNA damage repair pathways [28]. The evidence that PALB2 is critical for HR and functions as a breast and pancreatic susceptibility gene suggest that the role of the adaptor protein, PALB2, may be critical for BRCA1/2- mediated tumor suppression by physically linking BRCA1 to BRCA2. Since both germline and somatic mutations in *PALB2* and *BRCA1/2* genes were found in a significant proportion of pancreatic cancer cases [8], to understand those tumors better, it is also important to study whether tumors derived from defected function of PALB2, BRCA1 and BRCA2 are caused through a same mechanistic pathway by comparing similarities and differences between PALB2 and BRCA1/2 tumors. Thus, we generated mouse models of pancreatic cancer by inactivation of *Palb2*, *Brca1* or *Brca2* genes specifically in the pancreas and compared the resulting tumor latencies, histo-pathologies, anticancer drug responses and immune cell infiltration.

## Materials and methods

### Generation of murine models for pancreatic cancer

*Brca1*^*flox2/flox2*^[29], *Brca2*^*flox3-4/flox3-4*^ [30], and *Palb2* ^*flox2-3/flox2-3*^ (obtained from the laboratory of Dr. Bing Xia group, Cancer Institute of New Jersey) [31] were crossed to strains carrying *Kras*^*LSL-G12D/+*^, *Trp53*^*LSL-R270H*/+^ and *Pdx1-cre* (Strain number 01XL5, 01XM3 and 01XJ6 respectively, National Cancer Institute Frederick Mouse Repository) alleles to generate all the genotypes in this study. All transgenic animals were maintained on a mixed genetic background (129/B6). Genotyping results and primers are shown in supporting information—S1 Supporting Information and S1 Table respectively (S1 Supporting Information and S1 Table).

### Ethics statement

All animal studies were approved by the Ohio State University Institutional Animal Care and Use Committee (IACUC), and performed in compliance with the Guide for the Care and Use of Laboratory under protocols 2012A00000063 (PI-TL) and 2013A00000141 (PI-RS). Mice were housed under controlled conditions (12 hours light/dark cycle), given water and food ad libitum, and monitored every day by trained staff. When animals displayed any of early removal criteria or distress signs such as unresponsiveness, immobility, inability to feed or drink, excessive cachexia, dyspnea, lethargy, and rough hair coat we consulted with veterinary staff and euthanized the animals by CO2 inhalation followed by cervical dislocation. Tumor tissues were collected from euthanized mice for histological analysis.

### Histological analysis and immunohistochemistry (IHC)

Organs were fixed in a 10% formalin solution for 24-48hrs and stored in 70% ethanol. Formalin-fixed mouse pancreata and tumor tissue were embedded in paraffin, sectioned to obtain 4μm thick sections, and stained with hematoxylin and eosin (H&E) for histological analysis.

For immunohistochemistry, tissue sections were stained using a Bond Rx autostainer (Leica) [32]. Amylase (1:400, CST 3796), rat antibody-cytokeratin 19 (TROMA-III) (1:150, Developmental Studies Hybridoma Bank, University of Iowa), and CD3 (DAKO, A0452) were diluted in antibody diluent (Leica). Images were taken using the VECTRA^®^ Automated Quantitative Pathology Imaging system (PerkinElmer, Hopkinton, MA, USA). For estrogen receptor *alpha* (ER) (1:500, Santa Cruz, SC-542) and progesterone receptor (PR) (1:200, DAKO, A0098) staining, the manufacturer’s recommended protocol (Vector laboratories, VECTASTAIN ABC HRP Kit, PK-6101) was followed. For F4/80 (1:500, Invitrogen MF48000) immunofluorescence staining, deparaffinized and rehydrated slides were microwaved in sodium citrate solution. After blocking, primary antibody was incubated at 4 degree overnight and secondary antibody (Alexa Fluor 594, 1:500) for 1 hour at room temperature. Washed slides were mounted with mounting solution with DAPI.

### Establishment of primary pancreatic tumor cells

Isolated tumor tissue was cut into small pieces, trypsinized and neutralized. Cells were dispersed by passing through a syringe and needle several times and cultured until cell lines were established.

### Karyotype analysis

Cells were incubated in medium with or without DNA damaging agents (Mitomycin C (MMC) 40ng/ml or Olaparib 1μM) for 16hrs and treated with 0.05μg/ml KaryoMax colcemid (GIBCO) for 2hrs. Cells were harvested, incubated in pre-warmed 0.56% KCl solution for 30 minutes at 37 degree and fixed in Carnoy’s solution (75% methanol: 25% acetic acid). Metaphase spreads were prepared and stained in 0.5% Giemsa solution and analyzed on a Zeiss Axioskop microscope with a 100X objective under oil.

### Allograft assay

For subcutaneous injection of tumor cells into nude mice, cultured tumor cells (50~60% confluent) were harvested by trypsinization. After cell counting, cells were resuspended in 1% FBS in PBS solution. Mice were anaesthetized with 4% isoflurane, and 0.3X10^6^ cells/100μl were injected subcutaneously in the dorsal side of the upper hind limb of nude mice. 2 tumor cells per genotype were used, and each tumor cell line was injected to 3 animals for each treatment group. After 10 to 14 days, when tumor size was approximately 100mm^3^, MMC (5mg/kg, at day 1), Cisplatin (6mg/kg, at day 1 and 8) or vehicle control were injected to mice intraperitoneally. Tumor size was measured using calipers every 2–3 days after injection, and ulcerated tumors were excluded from data. When we observe weight loss exceeding >20% of body weight or allografted tumor diameter exceeding 1.5 cm we consulted with the veterinary staff and humanely euthanized the animals.

### Statistical analysis

Statistical analyses were performed using unpaired two-tailed Student’s t-test to compare sets of results from independent groups, and values of *p* < 0.05 was considered statistically significant. For Kaplan-Meier survival curves, significance was estimated with the log-rank test using Graph-Pad Prism 7 software.

## Results

### Pancreas-specific deletion of *Palb2*, *Brca1* or *Brca2* early in development results in smaller pancreata

**Fig 1:**
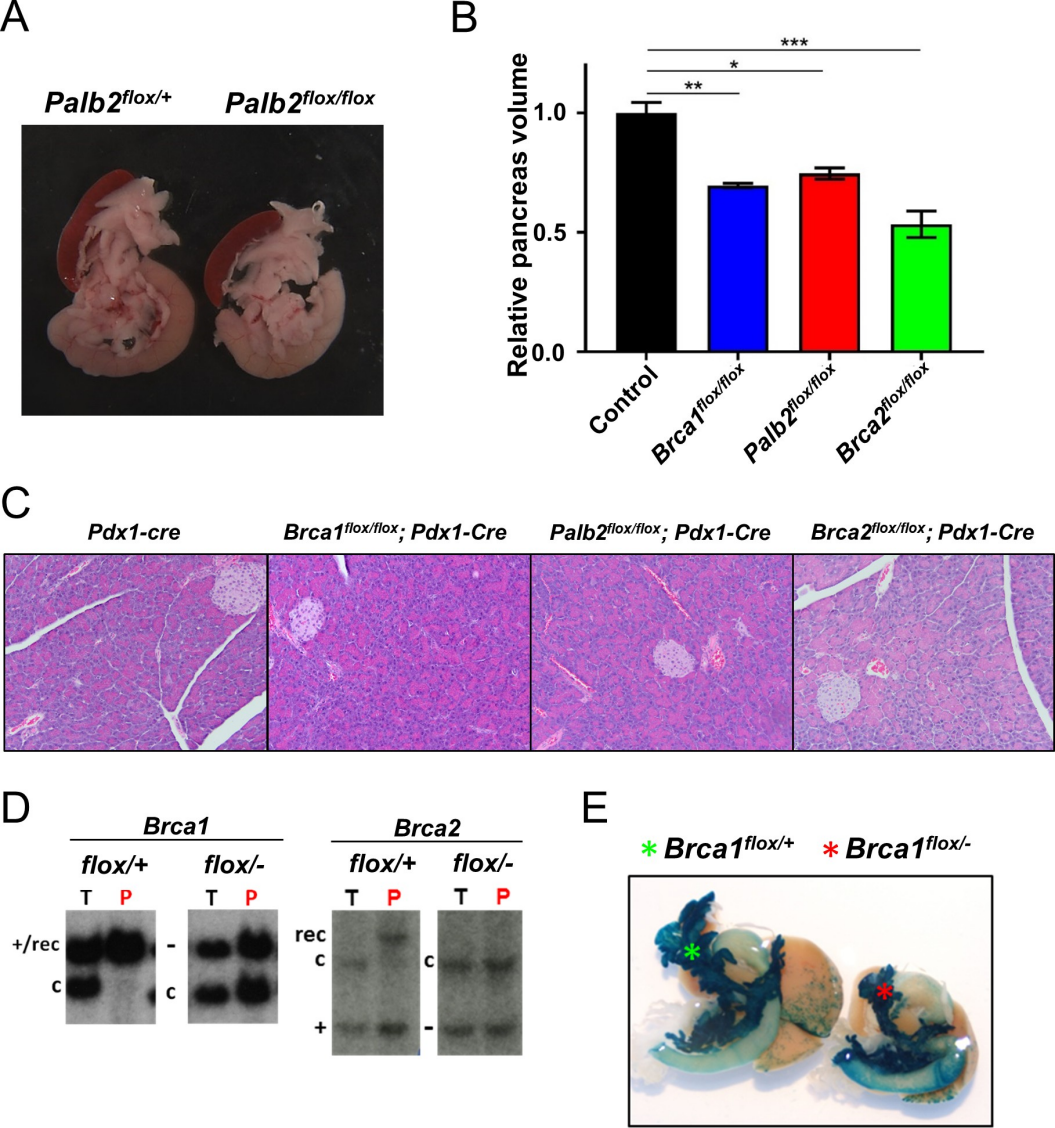
Pancreas-specific deletion of *Palb2*, *Brca1* or *Brca2* early in development results in smaller pancreata. (A) Smaller size of *Palb2*^*flox/flox*^; *Pdx1-Cre* pancreata compared to littermate control *Palb2*^*flox/+*^; *Pdx1-Cre* (B) Morphometry analysis on *Brca1*^*flox/flox*^; *Pdx1-Cre* (n = 3), *Palb2*^*flox/flox*^; *Pdx1-Cre* (n = 3) and *Brca2*^*flox/flox*^; *Pdx1-Cre* (n = 3) mouse tissues showed that *Brca1*-, *Palb2-* or *Brca2*- deleted pancreata volume were smaller than age matched controls (n = 9). (Means ± SEM; *P = 0.0105, **P = 0.0035, ***P = 0.0003 *vs* control tissue) (C) H&E (Hematoxylin and eosin) of *Brca1*^*flox/flox*^; *Pdx1-Cre*, *Palb2*^*flox/flox*^; *Pdx1-Cre* and *Brca2*^*flox/flox*^; *Pdx1-Cre* pancreata. (D) Southern blot analysis detected recombined *Brca1/2* conditional allele in pancreas of *Brca1/2*^*flox/+*^; *Pdx1-Cre* and *Brca1/2*^*flox/-*^; *Pdx1-Cre* animals (T: Tail genomic DNA and P: Pancreatic genomic DNA). Conditional allele from pancreas from *Brca1/2*^*flox/-*^; *Pdx1-Cre* animals remained unrecombined (+: Wild type allele, -: Null allele, c: floxed allele, and rec: Recombined floxed allele). (E) Detection of *Cre*-mediated recombination activity in pancreas using *Pdx1-Cre*; *Rosa26-LacZ* mice. Effective Cre recombination by LacZ staining was detected in pancreas of *Brca1*^*flox/flox*^; *Pdx1-Cre* and *Brca1*^*flox/-*^; *Pdx1-Cre* animals.

Whole body deletion of the mouse *Palb2* gene results in early embryonic lethality similarly to the *Brca1* and *Brca2* knock-out animals, indicating that all three tumor suppressor gene products, Palb2, Brca1 and Brca2 are essential for embryonic viability [33–35]. Hence, to circumvent the embryonic lethality and to study the role of these tumor suppressors in pancreatic development and malignant transformation, we specifically deleted *Palb2*, *Brca1* or *Brca2* in the pancreas only using the *Cre-LoxP* recombination technology. For this purpose, we used conditional null alleles of *Palb2* (*Palb2*^*flox2-3*^) [31], *Brca1* (*Brca1*^*flox2*^) [29] and *Brca2* (*Brca2*^*flox3-4*^) [30] in combination with the well characterized *Pdx1-Cre* transgene that has been extensively used for modeling pancreatic ductal adenocarcinoma (PDAC) in mice. The *Pdx1-Cre* transgene is expressed in the epithelial lineages of the embryonic pancreas (which includes both exocrine and endocrine lineages) and continues to be expressed throughout adulthood [36]. *PALB2*, *BRCA1* and *BRCA2* genes are essential for viability of normal cells; when normal cells are depleted of any of these gene products, they fail to proliferate and rapidly undergo senescence/apoptosis [33, 37, 38] Hence, we investigated whether pancreas-specific deletion of any of these genes would affect the pancreas development. Animals with conditional deletion of Palb2, Brca1 or Brca2 were born at expected Mendelian frequency and developed into healthy, fertile adults with overall body size and weight gain similar to the control littermates. In contrast, the pancreata among these animals of all three genotypes were significantly reduced in size *vs* the control littermate pancreata (Fig 1A and 1B). However, the overall histo-architecture of the pancreata was normal and not significantly different from those of control pancreata (Fig 1C). Next, we sought to confirm the recombination status of the conditional-null alleles of these genes in the affected pancreata by Southern blot analysis. For these recombination experiments, we utilized animals carrying only one floxed allele along with either one wild type for control animals, or one null allele for experimental animals, thus allowing comparison of recombination of equimolar amount of floxed allele between control *vs* experimental groups. In pancreata of heterozygous *Brca1*^*flox/+*^*;Pdx1-Cre* or *Brca2*^*flox/+*^*;Pdx1-Cre* mice, the “floxed” conditional allele were fully recombined. In contrast, in pancreata of *Brca1*^*flox/-*^*;Pdx1-Cre* and *Brca2*^*flox/-*^*;Pdx1-Cre* mice, the conditional allele remained unrecombined (Fig 1D). To verify that the lack of recombination of the floxed allele among the experimental animals is not due to the absence of *Pdx1-Cre* transgene expression in the affected pancreata, we further bred *Brca1*^*flox/-*^*;Pdx1-Cre* animals with the conditional *Rosa26R-LacZ* reporter allele, and observed the affected pancreata showed robust expression of the reporter lacZ as evidenced by X-gal staining (Fig 1E). These results indicate that the viability of pancreatic progenitor cells lacking Brca1 or Brca2 expression during pancreas development is severely compromised which in turn results in smaller pancreas size among *Brca1/2*^*flox/flox*^*;Pdx1-Cre* animals. We hypothesize that loss of these essential gene functions in normal pancreas cells likely causes either proliferative burst deficiency, senescence or apoptosis (or combination of theses) which are likely dependent on intact p53 tumor suppressor pathway activation. We postulate that likely a similar scenario occurs among pancreata of *Palb2*^*flox/-*^*; Pdx1-Cre* animals as well. Unfortunately, since we did not have access to mice carrying *Palb2*-null allele, we could not empirically test the recombination status of the *Palb2*-floxed allele in pancreata in a similar manner. *Palb2*^*flox/flox*^*;Pdx1-Cre*, *Brca1*^*flox/flox*^*;Pdx1-Cre* and *Brca2*^*flox/flox*^*;Pdx1-Cre* animals did not develop pancreatic cancer during the lifespans of these mouse strains, which could be because these mice still express the “floxed” conditional allele. Consistent with our findings, other group reported that inactivation of the *Brca2* gene alone without disruption of *p53* is not sufficient to promote tumor development [16].

### Concomitant expression of mutant *Kras*^*G12D*^ and *p53*^*R270H*^ cooperate with *Palb2*, *Brca1* or *Brca2* loss in the pancreas to promote PDAC tumorigenesis

PALB2, BRCA1 and BRCA2 proteins are involved in DNA damage repair, primarily through their roles in homologous recombination (HR). PALB2 is a linker protein physically and functionally connecting BRCA1 and BRCA2 during HR [24]. In the context of an intact p53-induced DNA damage checkpoint, the accumulation of chromosomal abnormalities as a result of loss of PALB2, BRCA1 and BRCA2 culminates in cell death. Hence, for cells that have lost PALB2, BRCA1 or BRCA2 to undergo neoplastic transformation, first, they would have to overcome the DNA damage induced checkpoint by inactivation of p53. As mentioned above, pancreatic progenitor cells that lack Palb2, Brca1 or Brca2 functions are eliminated during embryonic development, presumably via p53-induced apoptosis. Most *p53* mutations identified from tumors are missense and typically affect the DNA binding domain. The R273H mutation (R270H in the mouse) is one of the hot-spot mutations in the human *p53* gene [39]. Previous reports showed that the mutant p53 R270H protein has a dominant-negative inhibition effect of wild type p53. More specifically, heterozygous mutant allele of *p53*^*R270H*^ delayed transcriptional activation of its downstream target genes and inhibited *p53* dependent apoptosis [40]. Therefore, we chose the “conditional knock-in” LSL-*p53*^*R270H*^ mutant allele, LSL-*p53*^*R270H*^, for our pancreatic tumor mouse models.

Activating mutations in the *Kras* gene (e.g., *Kras*^*G12D*^) are the most frequent mutations found in human PDACs with some studies reporting a prevalence rate as high as 90% [41, 42]. Also, in agreement with the hypothesis that *Kras*^*G12D*^ mutations are likely to be involved in PDAC initiation, these mutations are frequently found in early precursor lesions of PDAC, such as pancreatic intraepithelial lesions (PanINs) [42]. Hence, we decided to delete *Palb2*, *Brca1* or *Brca2* concomitant with mutant *Kras*^*G12D*^ expression—using a “conditional knock-in” mutant allele, LSL-*Kras*^*G12D*^, and henceforth simply referred as *Kras*^*G12D*^ [43].

**Fig 2:**
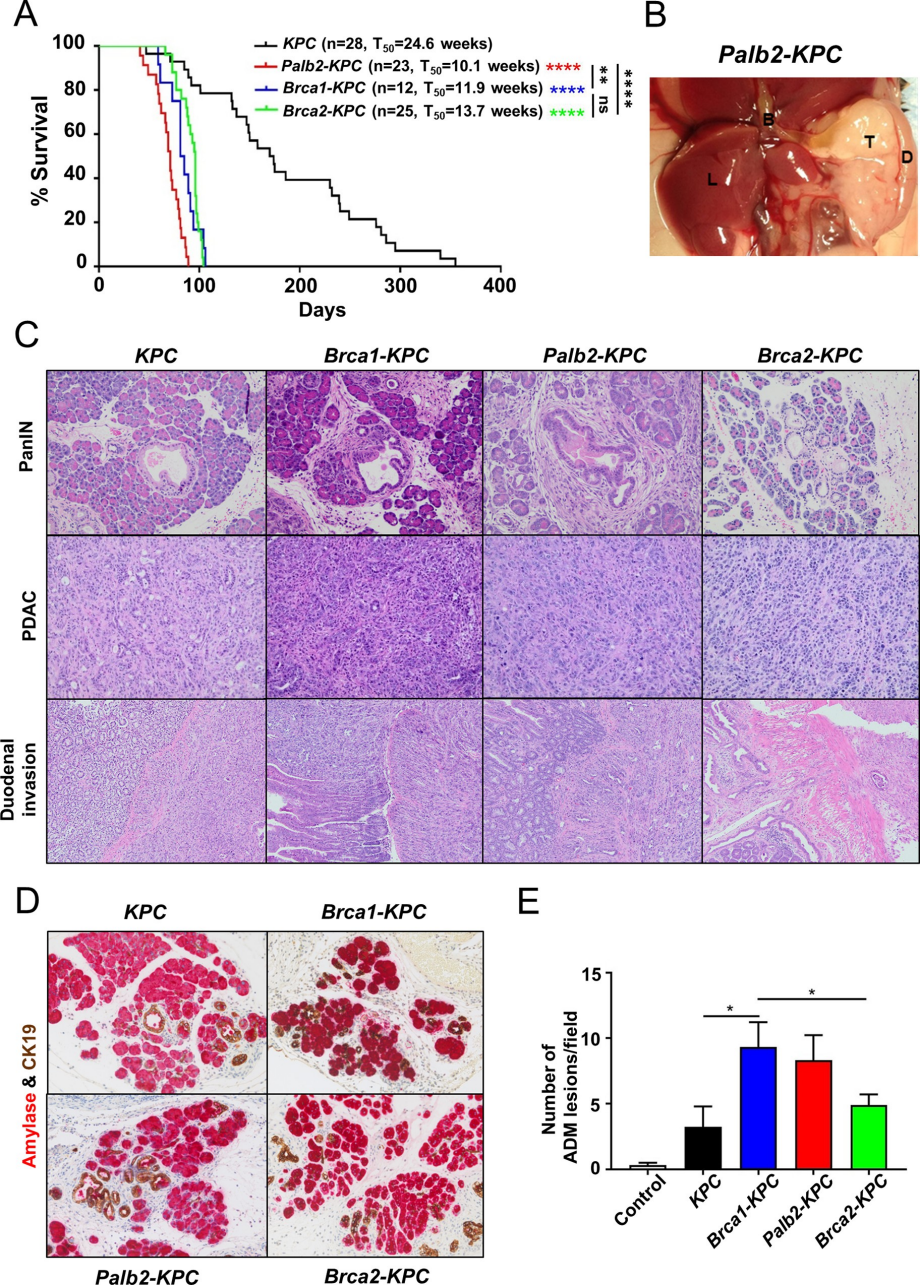
Concomitant expression of mutant *KrasG12D* and *p53R270H* cooperates with *Palb2*, *Brca1 or Brca2* loss in pancreatic ductal cells to promote PDAC tumorigenesis. (A) Kaplan-Meier survival curves of *KPC*, *Brca1-KPC*, *Palb2-KPC* and *Brca2-KPC*. Higher rates of death were observed in *Brca1-KPC*, *Palb2-KPC* and *Brca2-KPC* compared with *KPC* (****P<0.0001 compared with KPC). *Palb2-KPC* animals showed slightly, but significantly worse survival rates than *Brca1-KPC* and *Brca2-KPC* (****P<0.0001, **P = 0.0022). (B) Gross appearance of pancreatic tumor of *Palb2-KPC* mouse. Large solid tumor in the head of pancreas growing into bile duct (T: tumor, L: Liver, B: Bile duct and D: Duodenum). (C) H&E (Hematoxylin and eosin) analysis of the histopathology of *KPC*, *Brca1-KPC*, *Palb2-KPC* and *Brca2-KPC* tissues (D) Detection of acinar ductal metaplasia (ADM) lesions by immunohistochemical double staining of amylase and cytokeratin 19. (E) Quantification of acinar ductal metaplasia (ADM) lesions in the mouse strain pancreata (Means ± SEM; *P<0.05).

It is known that the Palb2, Brca1, or Brca2 proteins, with p53, synergistically suppresses tumor development, and mutations in the *p53* gene are common in pancreatic cancer [16, 20, 31]. Therefore, we generated the *Palb2-KPC* (*Palb2*^*flox/flox*^*; Kras*^*G12D*^*; p53*^*R270H/+*^*;Pdx1-Cre*), Brca1-*KPC* (*Brca1*^*flox/flox*^*; Kras*^*G12D*^*; p53*^*R270H/+*^*;Pdx1-Cre*), Brca2-*KPC* (*Brca2*^*flox/flox*^*; Kras*^*G12D*^*; p53*^*R270H/+*^*; Pdx1-Cre)*, or *KPC* (*Kras*^*G12D*^*; p53*^*R270H/+*^*; Pdx1-Cre)* animals, and monitored tumor development. In contrast to *KPC* animals (n = 28, T_50_ = 24.6 weeks), *Palb2-KPC*, *Brca1-KPC or Brca2-KPC* respectively developed PDAC with a much shorter pancreatic tumor-free median survival of 10.1, 11.9 and 13.7 weeks, respectively (Fig 2A). Like *KPC* animals, these animals when moribund presented with swollen abdomen (upon necropsy, we often found hemorrhagic ascitic fluid within the peritoneal cavity) and severe lack of body fat. *Palb2-KPC* mice became moribund slightly sooner than *Brca1-KPC or Brca2-KPC* animals. Most of the *Palb2-KPC* mice have tumor developing in the head of the pancreas where they are more likely to grow into the bile-duct (Fig 2B). 21 of 23 *Palb2-KPC* animals had a solid tumor in the head of the pancreas and in 14 cases, the tumor caused blockage of the bile-duct causing jaundice. Based on the survival data, we could conclude that concomitant loss of *p53* and *Palb2*, *Brca1* or *Brca2* tumor-suppressor functions cooperate to dramatically augment tumorigenic potential of oncogenic mutations, such as *Kras*^*G12D*^.

We compared the histo-pathology of the pancreatic tumors that developed among *Palb2-KPC*, *Brca1-KPC* and *Brca2-KPC* to those of *KPC* animals. Among all four genotypes, we found precursor pancreatic intraepithelial neoplasia (PanIN) lesions at various stages of atypia that progressed into full-blown PDAC (Fig 2C). With the progression of the disease, these PDACs eventually engulfed the entire pancreas and began to invade the nearby organs within the peritoneal cavity, such as the duodenum, spleen and kidney (Fig 2C). Besides classical PanINs that are thought to originate within the epithelial lining of the pancreatic ductules, Acinar-to-ductal metaplasia (ADM) within the acinar population in the pancreas can also result in PDAC precursor lesions [42, 44]. We observed ADM among all four genotypes as determined by immuno-histochemical (IHC) analysis of amylase and cytokeratin 19 (CK19) (Fig 2D). ADM incidence was significantly elevated in *Brca1-KPC* tumors compared to *KPC* tumors, but not in *Brca2*-*KPC* tumors. *Palb2-KPC* showed intermediate levels of ADM lesions between those of *Brca1-KPC* and *Brca2*-*KPC* tumors (Fig 2E).

### Pancreatic cystic lesions resembling MCNs are common to *Palb2* and *Brca1*-mutant animals

**Fig 3:**
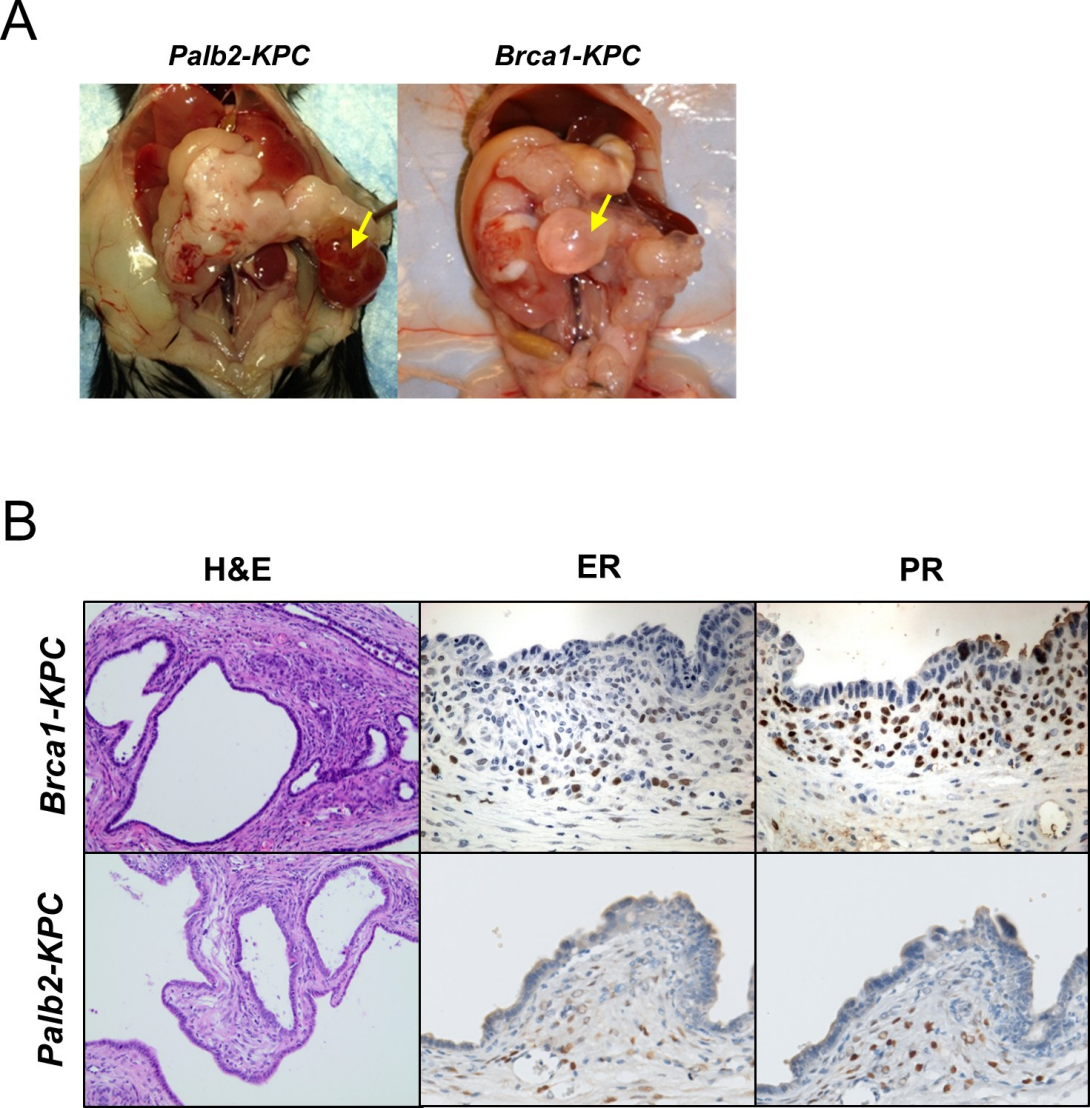
Pancreatic cystic lesions resembling MCNs are unique to *Palb2* and *Brca1*-mutant animals. (A) Gross morphology photographs of primary tumors in pancreas of *Brca1-KPC* and *Palb2-KPC*. Yellow arrows indicate the large pancreatic cysts. (B) H&E staining of cystic lesions in *Brca1-KPC* and *Palb2-KPC* pancreatic tumors. Immunohistochemistry for ER and PR showed epithelial cells associated with ovarian-like stroma.

As described above, *Brca1-KPC* animals developed classical PanINs and ADMs that eventually progressed to PDAC. In addition, the majority of *Brca1-KPC* presented with cysts that were grossly visible upon dissection (Fig 3A). These cystic lesions were frequently numerous and multi-lobular, some as large as 2 to 3 cm in size and yielded as much as a few milliliters of serous fluid with hemorrhagic components and cellular debris. The epithelial lining of some of these cysts displayed atypia. These cystic lesions often resembled mucinous cystic neoplasms (MCN) (Fig 3B); they were often found in the tail and body of pancreas and were circumscribed by ovarian-like stroma with wavy nuclei and expressed steroid hormone receptors, namely estrogen receptor (ER) and progesterone receptor (PR) [45, 46]. Among *Palb2-KPC* animals, 6 of 23 (26%) animals presented these cystic lesions that were surrounded by ER and PR positive ovarian-like stroma (Fig 3B).

Unlike *Brca1-KPC* and *Palb2-KPC* animals, we did not observe clearly visible cystic lesions in the *KPC* and *Brca2-KPC* animals. Therefore, *Palb2-KPC* animals present an intermediate phenotype regarding the pancreatic neoplasia spectrum, some presenting a phenotype similar to *Brca1*-*KPC* (presence of large cystic lesions), while others are more similar to *Brca2-KPC* animals.

### Primary tumor cells derived from *Palb2-KPC*, *Brca1-KPC* and *Brca2-KPC* pancreatic tumors exhibit hypersensitivity to DNA damaging agents

**Fig 4:**
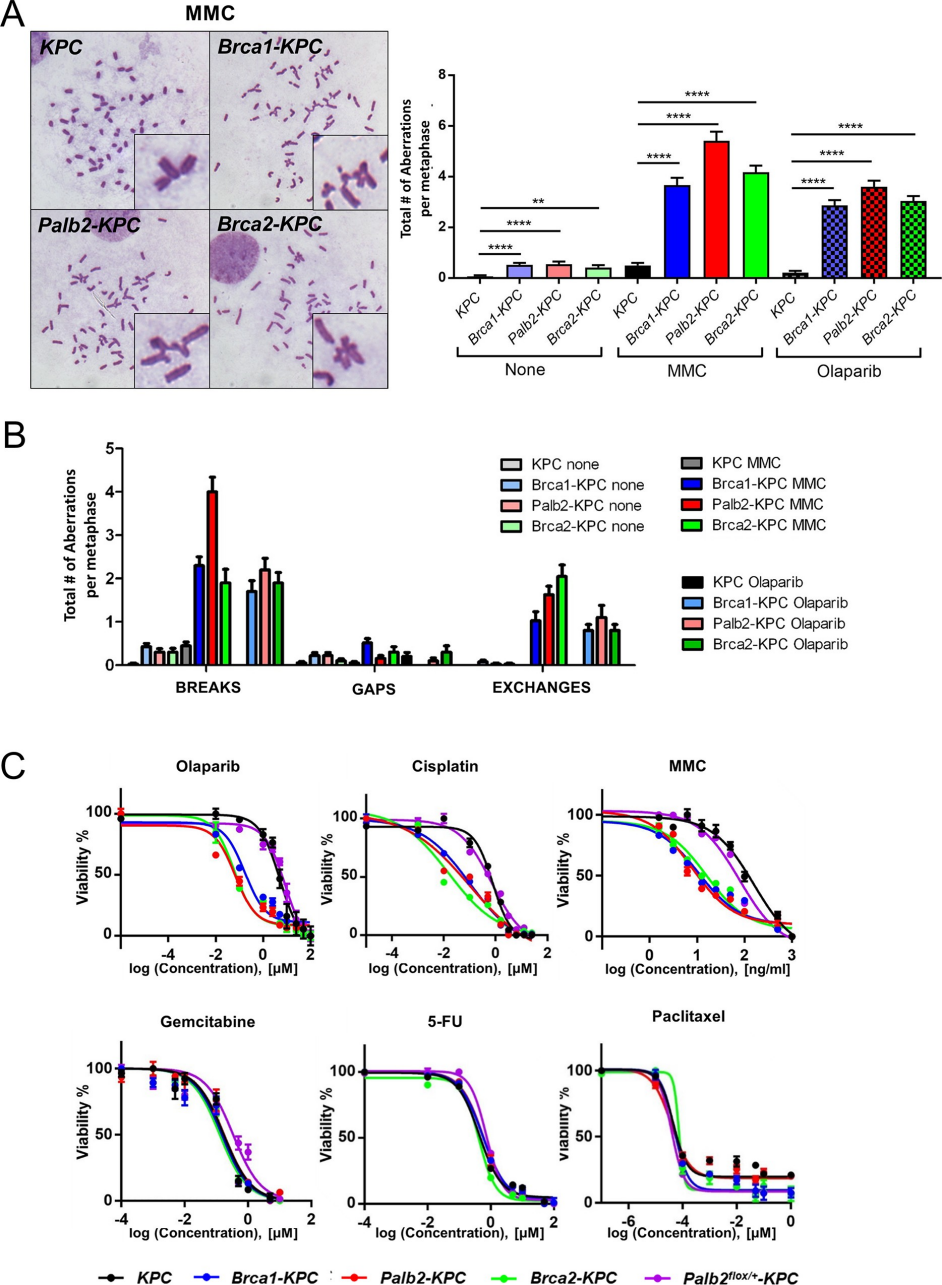
Sensitivity to DNA damaging agents of primary tumor cells from *Palb2-KPC*, *Brca1-KPC* and *Brca2-KPC* solid tumors detected by karyotype analyses. (A) *Palb2-KPC*, *Brca1-KPC* and *Brca2-KPC* pancreatic tumor cells showed increased sensitivity *vs KPC* tumor cells, to MMC and Olaparib. Representative images of metaphase spreads from *Palb2-KPC*, *Brca1-KPC* and *Brca2-KPC* pancreatic tumor cells treated with MMC. Total chromosome aberrations in individual tumors following drug treatments (Means ± SEM; ****P<0.0001, **P<0.0011). (B) Responses to specific drug treatments of cells from specific mouse strain tumors was measured by MTT assay. Exposure to each drug was for 72 hrs before the MTT assays were performed. The data are from 6 replicates of 2 tumor cells per genotype. For the Olaparib treatment, due to its short half-life, medium with fresh drug was changed every 24 hrs. (Mean ± SEM).

As noted above, PALB2, BRCA1 and BRCA2 play important roles in DNA damage repair, mainly in the HR pathway [47]. Therefore, deletions or hypomorphic mutations in these genes cause genome instability often characterized by gross chromosomal abnormalities, and thus resulting in cells with hypersensitivity to DNA damaging drugs such as interstrand cross-linking agents (ICL agents) (e.g., Mitomycin C and Cisplatin) and poly ADP ribose polymerase inhibitors (PARP inhibitors) (e.g., Olaparib) [48–52]. Thus, we expected chromosomal instability and hypersensitivity of primary tumor cells isolated from *Palb2-KPC*, *Brca1-KPC*, *or Brca2-KPC* pancreatic tumors to DNA damaging agents. To verify this, we generated multiple cell lines from tumors of each mouse model (*KPC*, *Palb2-KPC*, *Brca1-KPC*, *Brca2-KPC*) and performed metaphase karyotype analyses after confirmed recombination of “floxed” alleles (S1 Supporting Information). All primary pancreatic tumor cell lines expressed CK19, indicating they were originated from ductal cells (S2 Supporting Information). As expected, metaphases of *Palb2*-, *Brca1*- or *Brca2*- deleted cells showed significantly increased numbers of chromosomal aberrations even without treatment with DNA damaging drugs compared to *KPC* control cells. After Mitomycin C (MMC) or Olaparib treatment, the numbers of chromosomal abnormalities of *Palb2-KPC*, *Brca1-KPC*, or *Brca2-KPC* cells were dramatically increased compared to metaphases of *KPC* control cells. Metaphases from these cells had various types of chromosomal aberrations including breaks, gaps and exchanges (Fig 4A). We also compared drug IC50s of tumor cells from each group. For the IC50 measurement, *Palb2*^*flox/+*^-*KPC* cells were included to assess haploinsufficiency in DNA damaging drug sensitivity. Consistent with karyotype results, IC50 values for DNA damaging agents are much lower in *Palb2-KPC*, *Brca1-KPC*, and *Brca2-KPC* cells, *vs KPC* cells, whereas *Palb2*^*flox/+*^-*KPC* cells behave like *KPC* cells, indicating that one wild type copy of *Palb2* is sufficient for DNA repair caused by MMC, Cisplatin and Olaparib. In contrast to the response to DNA damaging agents, we did not observe differences of sensitivity to other classes of chemotherapy drugs such as Paclitaxel, Fluorouracil (5-FU) and Gemcitabine among the 5 different genotypes (*KPC*, *Palb2*^*flox/+*^-*KPC*, *Palb2-KPC*, *Brca1-KPC*, and *Brca2-KPC*) (Fig 4B and Table 1)

**Table 1:** IC_50_ values.

	Olaparib [95% CI] (μM)	Cisplatin [95% CI] (μM)	MMC [95% CI] (ng/ml)	Gemcitabine [95% CI] (μM)	5-FU [95% CI] (μM)	Paclitaxel [95% CI](mM)
***KPC***	5.221 [3.439–7.978]	0.775 [0.657–0.908]	158.0 [115.3–224.6]	0.1705 [0.132–0.218]	0.441 [0.376–0.514]	0.041 [0.027–0.063]
***Brca1-KPC***	0.170 [0.100–0.341]	0.103 [0.068–0.158]	10.71 [7.721–15.14]	0.150 [0.113–0.196]	0.561 [0.451–0.689]	0.044 [0.031–0.064]
***Palb2-KPC***	0.067 [0.035–0.122]	0.098 [0.046–0.273]	7.837 [6.018–10.34]	0.175 [0.135–0.225]	0.574 [0.473–0.691]	0.037 [0.025–0.055]
***Brca2-KPC***	0.061 [0.036–0.104]	0.016 [0.009–0.029]	15.70 [11.69–21.17]	0.129 [0.108–0.153]	0.428 [0.363–0.511]	0.042 [0.028–0.063]
***Palb2***^***flox/+***^***-KPC***	8.783 [6.161–12.64]	0.700 [0.522–0.981]	78.36 [65.20–94.60]	0.339 [0.244–0.463]	0.734 [0.655–0.814]	0.035 [0.027–0.044]

IC_50_ values were determined using MTT assay after 72 h drug treatment of each indicated drug. Experiments were done in triplicate or quadruplicate ± SEM, and all values are averages of replicates from two different cell lines relative to cell viability values without treatment. IC_50_ values were calculated by nonlinear regression using Graph-Pad Prism 7 software.

### Interstrand crosslinking agents inhibit *Palb2-KPC*, *Brca1-KPC* and *Brca2-KPC* tumor growth *in vivo*

**Fig 5:**
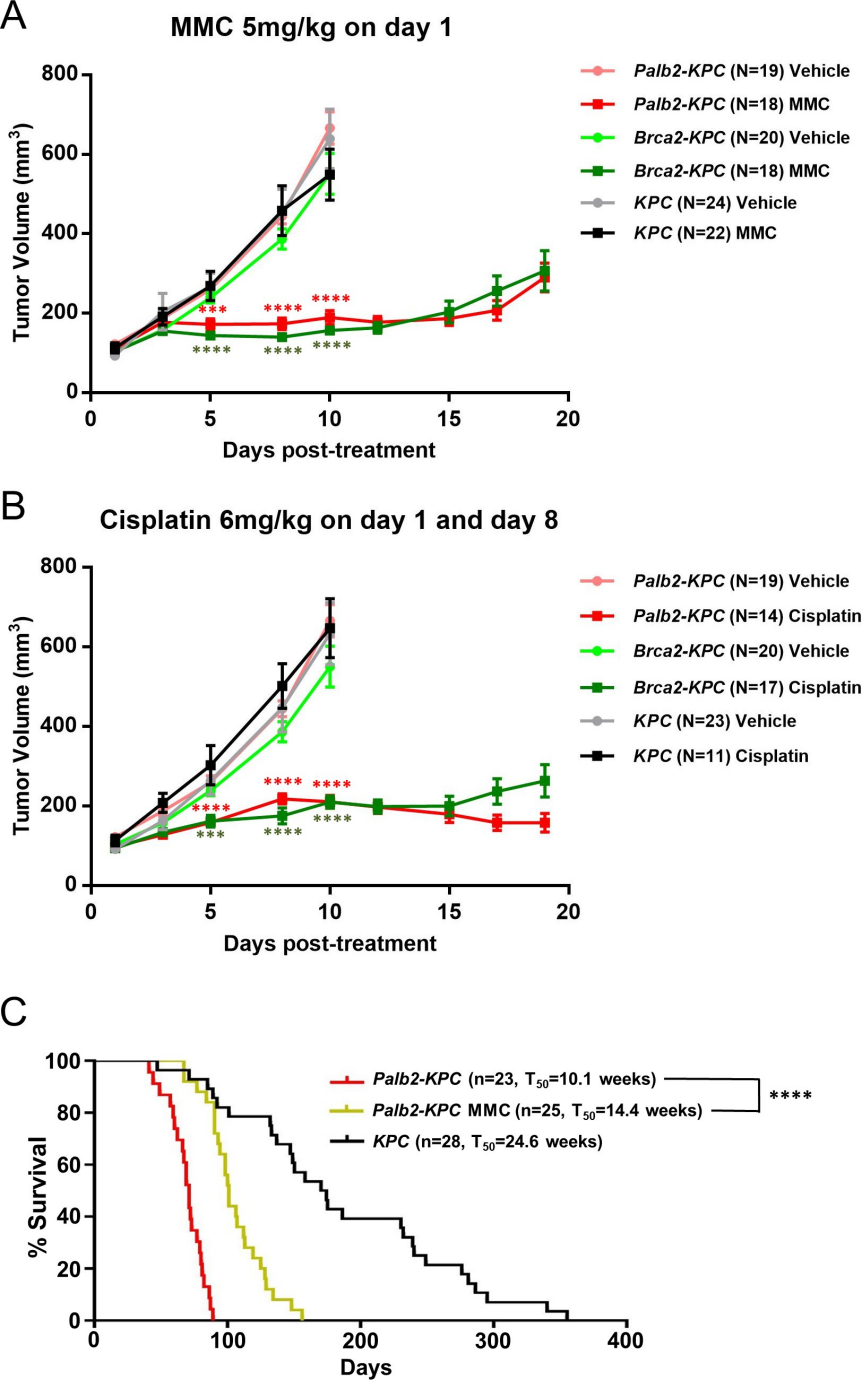
Interstrand crosslinking agents inhibit *Palb2-KPC* and *Brca2-KPC* tumor growth *in vivo*. (A, B) Growth curves of allograft tumors in nude mouse models. MMC and Cisplatin treatment inhibited growth of *Palb2-KPC*, *Brca1-KPC* and *Brca2-KPC* tumors (Means ± SEM; ***P<0.0005, ****P<0.0001 *vs* vehicle treated group using). (C) Kaplan-Meier survival curve of *Palb2-KPC* with or without MMC. MMC treatment prolonged survival of *Palb2-KPC* animals (5mg/kg, every 3 weeks injection from 3~6 weeks of age) (****P<0.0001 compared with the untreated group).

As described above, *Palb2*-, *Brca1*- or *Brca2*- deleted tumor cells exhibit dramatically elevated sensitivity to DNA damaging drugs compared to *KPC* cells *in vitro*. Next, we tested whether this *in vitro* hypersensitivity to DNA damaging drugs can translate into tumor growth retardation *in vivo*. For this purpose, we generated subcutaneous allografts in immune-compromised athymic nude mice with above mentioned *Palb2-KPC*, *Brca2-KPC* and *KPC* pancreatic tumor cells and treated tumor-bearing mice with either MMC, or Cisplatin, or vehicle. Regardless of genotype, there was no significant difference in tumor growth among vehicle treated groups. Consistent with the *in vitro* results, MMC or Cisplatin treatment dramatically retarded growth of *Palb2-KPC* and *Brca2-KPC* tumors *vs* vehicle-treatment (Fig 5A and 5B). In contrast, at the same drug dose and regimen, *KPC* tumor growth was indistinguishable from vehicle-treated tumors. We further confirmed these *in vivo* drug-efficacy observations by treating *Palb2-KPC* animals that spontaneously develop PDAC with MMC on a weekly basis. Starting at 3 weeks of age, when these animals were treated with MMC for three weeks, the MMC-treatment significantly increased (P<0.0001) median survival to 14.4 weeks (MMC, T_50_ = 14.4 weeks *vs* untreated, T_50_ = 10.1 weeks) compared to vehicle-treated animals (Fig 5C).

### Immune cell infiltration increase in *Palb2-KPC* and *Brca1-KPC* tumors but not in *Brca2-KPC tumors*

**Fig 6:**
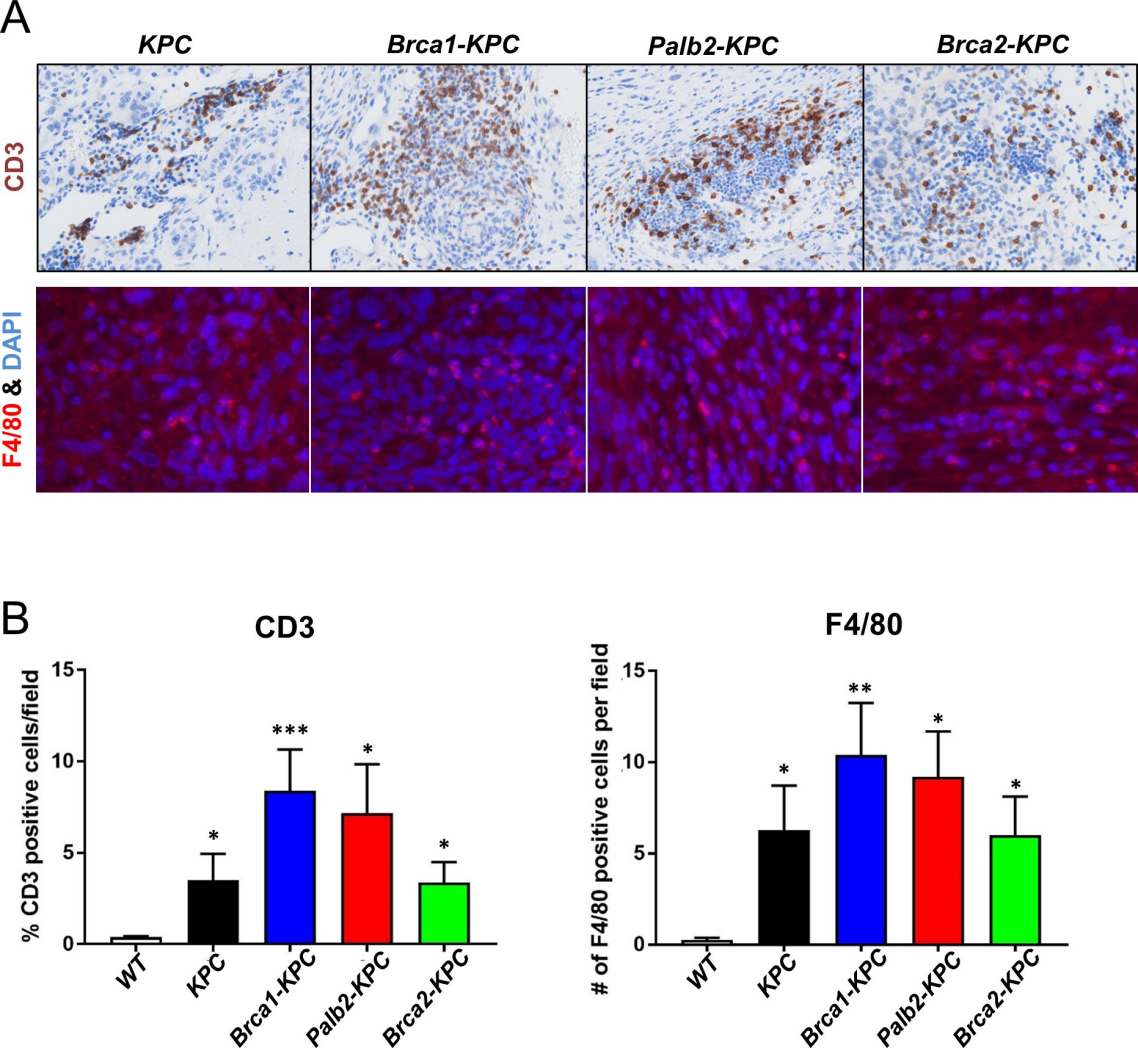
Infiltrating immune cells are increased in *Palb2-KPC* and *Brca1-KPC* tumors but not in *Brca2-KPC*. (A) Representative images of immunohistochemistry and immunofluorescence staining of CD3 and F4/80 in *KPC*, *Brca1-KPC*, *Palb2-KPC* and *Brca2-KPC* tumors. (B) Quantification of CD3 and F4/80 staining per field showed elevated CD3 positive T cells and F4/80 positive macrophages observed in *Brca1-KPC* and *Palb2-KPC* pancreatic tumors compared with *KPC* and *Brca2-KPC* (Means ± SEM; *P<0.05, **P = 0.0005, ***P = 0.0003 *vs*. wild type (WT) pancreata).

Although immunotherapy has emerged as a powerful cancer-targeting therapy, it has met with limited success in PDAC because of its immune suppressive features [53, 54]. Infiltration of immune cells such as effector T cells is necessary for the response to immune therapy. To determine the level of tumor-infiltrating immune cells, we examined CD3^+^ T cells and F4/80^+^ macrophages in tumors from *KPC*, *Palb2-KPC*, *Brca1-KPC*, and *Brca2-KPC* animals. All four groups of tumors showed increased T cells in PDAC as well as macrophages compared to normal pancreata. *Brca1-KPC* and *Palb2-KPC* tumors were highly infiltrated with CD3^+^ T cells and F4/80^+^ macrophages, although the difference of immune cells recruitments between groups was not significant (Fig 6A). Previous data from Mace *et al*. have shown that *Brca2*-*KPC* mice are resistant to single agent PD-L1 antibody blockade immunotherapy [55]. This data suggests that *Brca1-* and *Palb2-* deleted tumors might show different levels of resistance for immunotherapy.

## Discussion

It is well-known that germline mutations in *BRCA1* or *BRCA2* genes increase risks of breast and ovarian cancer, as well as pancreatic cancer [5, 6, 56, 57]. As *PALB2* mutations also have been observed in families with breast, ovarian and pancreatic cancer *like BRCA1/2*, the *PALB2* gene has also been recognized as a tumor susceptibility gene [4, 7]. Proteins encoded by these three tumor susceptibility genes (*PALB2*, *BRCA1*, *BRCA2*) cooperate in the BRCA pathway by forming a complex, which is essential for HR repair. In the complex, PALB2 physically interacts with and connects BRCA1 and BRCA2 [24]. The fact that mutations in those genes predispose to particular types of cancer and the BRCA1-PALB2-BRCA2 complex plays an important role in HR, suggest that they likely act together for their tumor suppression functions. In support of this thought, mutations that disrupt formation of the BRCA1-PALB2-BRCA2 complex have been found in breast, ovarian and pancreatic cancers [58, 59]. Thus, we hypothesized that the role of the PALB2 linker protein may be critical for BRCA1/2-mediated tumor suppression. To test this hypothesis, we generated *Palb2-*, *Brca1*- and *Brca2*- deficient pancreatic tumor mouse models and have shown that loss of *Palb2* cooperates with mutant *p53*^*R270H*^ and *Kras*^*G12D*^ in the development of PDACs, similarly to the *Brca1*- and *Brca2*-*KPC* mouse pancreatic tumor models, but *Palb2-*, *Brca1*- and *Brca2*-*KPC* tumors were not identical.

As for *Brca1* and *Brca2*, whole-body knock out of *Palb2* in mice leads to embryonic lethality, and the growth arrested *Palb2-*, *Brca1*- or *Brca2*- embryos commonly showed activation of p21 [33, 37, 38], indicating that the full-length gene products are essential for cellular viability and proliferation in the early embryo. In this study, we observed that pancreas specific deletion of *Palb2*, *Brca1* or *Brca2* results in decreased organ size. More importantly, recombination of the floxed alleles was not detectable in *Brca1/2*^*flox/-*^*;Pdx1-Cre* animals, indicating that *Palb2*, *Brca1* or *Brca2* deficient pancreatic progenitor cells during early embryonic development could not survive long term either due to proliferation defect, senescence or apoptosis or combination of these among them. Thus, for these defective cells to survive and proliferate, there is immense pressure on these cells to acquire secondary mutations in other genes that aid in overcoming proliferative arrest, senescence and apoptosis, e.g., *p53* which can lead to uncontrolled cell growth and proliferation and thus eventually cancer. We also questioned how pancreas could be developed if all progenitors that are null for one of these genes are not present in the pancreas during early development. It has been reported that *Pdx1-Cre* mediated gene recombination is variable because of mosaic Cre recombinase activity [60]. Therefore, it is likely that the organ will repopulate from stem cells in which recombination of some conditional alleles has not occurred.

Rowley *et al*. has reported that deletion of Brca2 in pancreas that concomitantly expresses mutant KrasG12D inhibits tumor development, and the few pancreatic tumors that form spontaneously acquire p53 mutations [16]. Indeed the few animals in our study with deletion of Palb2, Brca1 or Brca2 with concomitant KrasG12D mutation (i.e., Palb2-, Brca1- or Brca2-KC animals) showed accumulation of p53 protein in the pancreas consistent with Rowley et al observations (unpublished observations). Unfortunately, the numbers of animals of these genotypes were insufficient for us to either support or refute Rowley *et al*. study conclusions regarding tumor latency and prevalence. Next, we hypothesized that preventing growth inhibition and apoptosis induced by p53 activation upon loss of Palb2, Brca1 or Brca2 functions may allow resulting genomic instability to accumulate in KrasG12D mutant pancreata. This, in turn, could help to promote and accelerate PDAC progression in these triple-mutant animals. In this study, we showed that concomitant expression of *Kras*^*G12D*^ and *p53*^*R270H*^ mutations with *Palb2*, *Brca1* or *Brca2* deletion accelerated pancreatic tumor development. These results are consistent with Rowley et al. data for *Brca2* mutation in mouse model of pancreatic cancer in which *Brca2* and *p53* mutations synergize to accelerate pancreatic tumor development. *Palb2*-*KPC* mice developed PDAC and became moribund with shorter latency than *Brca1-KPC or Brca2-KPC* mice, possibly because >90% of *Palb2-KPC* tumors originated in the head of the pancreas obstructing the bile duct. We have seen a similar phenotype with *Brca2*^*Δex3*^*-KPC* mice (unpublished observations) where at least 70% of animals presented with jaundice due to bile-duct blockage by tumors in the head of the pancreas. It has been reported that patient derived missense mutations located in Exon3 of *BRCA2* abolished or dramatically reduced the PALB2-BRCA2 interaction [21]. Therefore, the specific tumor location in the head of the pancreas in *Palb2-KPC* and *Brca2*^*Δex3*^*-KPC* mice might be due to ablation of PALB2-BRCA2 interaction. Further study is required to prove this hypothesis.

**Fig 7:**
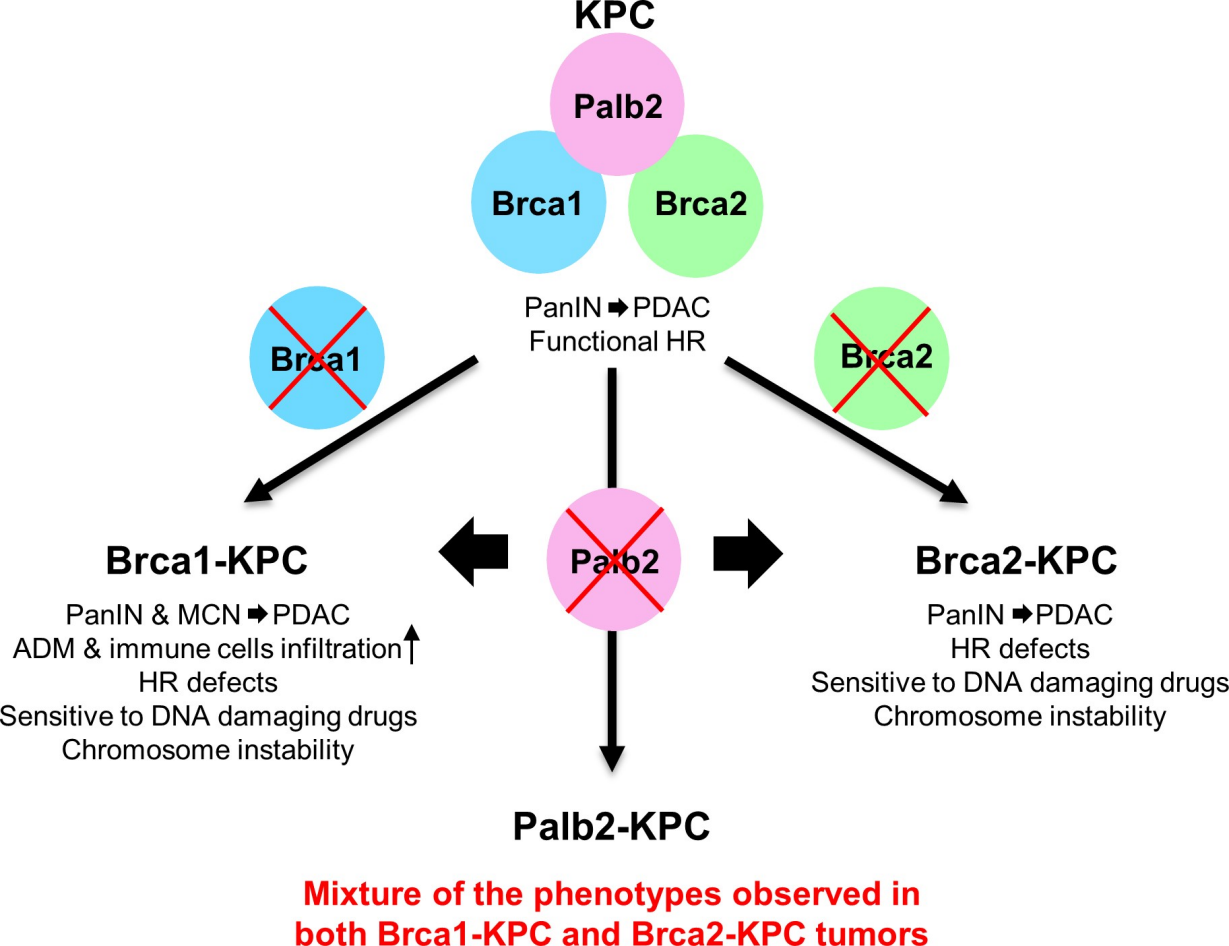
Graphical summary of phenotypes in *Brca1-KPC*, *Palb2-KPC* and *Brca2-KPC* pancreatic cancer mouse models. *Palb2*- deficient pancreatic tumors exhibited *Brca1*- deficient tumor like- or *Brca2-* deficient tumor like- features in terms of the presence of different PDAC precursor lesions and the intermediate levels of ADM lesions and immune cells infiltrations, possibly due to its role as a linker between Brca1 and Brca2.

Unexpectedly, *Brca1-KPC* and *Brca2-KPC* tumors were quite different in terms of formation of cystic lesions. While almost all *Brca1-KPC* animals developed large cysts with PanIN derived PDAC, no apparent cystic lesions were observed in *Brca2-KPC* and *KPC* mice. Similar to our finding, a study has shown increased incidence of abnormal pancreatic imaging findings in BRCA1 and BRCA2 mutation carriers, in which pancreatic cysts were found only in BRCA1 mutation carriers (2 out of 14), not BRCA2 mutation carriers (0 out of 6), although the number of patients was limited [61]. Interestingly, *Palb2-KPC* tumors showed a mixture of *Brca1-KPC* and *Brca2-KPC* tumor phenotypes regarding the presence of cysts. 6 of 23 *Palb2-KPC* tumors had large cysts with PDAC, which closely resembled the *Brca1-KPC* tumors. However, the remaining tumors showed no large cystic lesions similarly to *Brca2-KPC* or *KPC* tumors. These results suggest that pancreatic cysts in patients with BRCA1 or PALB2 mutations require a detailed autopsy and analysis. The large size of cystic lesions observed in *BRCA1* and some *PALB2-KPC* animals might be easily detected by abdominal imaging. The observed dichotomy in precursor lesions between the *Brca1*-deficient and *Brca2*-deficient tumors and the mixture of both in *Palb2* deficient tumors might provide insights into mechanisms underlying the higher pancreatic cancer incidence and aggressiveness in BRCA2 *vs* BRCA1 mutation carriers. Moreover, we observed increased ADM lesions and immune cells infiltrations such as CD3^+^ and F/480^+^ cells in *Brca1-KPC*, but not in *Brca2-KPC*, compared to *KPC* tumors. *Palb2-KPC* tumors showed intermediate phenotypes between *Brca1-KPC* and *Brca2-KPC*. The mixed phenotype of *Palb2-KPC* tumors could be due to the role of PALB2, as a linker protein between BRCA1 and BRCA2, in BRCA1/2 mediated tumor suppression (Fig 7).

To confirm HR deficiency in tumor cells deficient *Palb2*, *Brca1* or *Brca2* genes, drug sensitivity was determined by karyotype analysis and IC50 determination. In this study, using primary tumor cell lines, we measured the degree of sensitivity to different chemotherapeutic drugs. *Palb2-*, *Brca1-* or *Brca2-* deficient tumor cells in comparison to *KPC* or *Palb2*^*flox/+*^*-KPC* tumor cells are exquisitely sensitive to DNA damage inducing agents, including Olaparib, MMC and Cisplatin. However, the cells from the 5 different tumor genotypes did not show differential sensitivity to non-DNA damage-inducing drugs (5-Fluorouracil, Paclitaxel or Gemcitabine) that are routinely used in PDAC therapy regimens. Based on the *in-vitro* experiments, we also confirmed efficacy of MMC and cisplatin treatment *in vivo*. The DNA damaging drugs also inhibited tumor growth *in vivo*, resulting in decreased tumor burden and prolonged survival of treated animals. These preclinical results indicate that DNA damaging agents are effective and could be particularly useful in the treatment of *PALB2-*, *BRCA1-* or *BRCA2-* deficient pancreatic tumors, which is reminiscent of human cancers defective for ‘BRCAness’ [62, 63]. Although the DNA damaging agents have shown clinical efficacy in *PALB2-*, *BRCA1-* or *BRCA2-* cancers, tumor cells often develop resistance to these drugs, with tumor recurrence and progression. Therefore, it may be especially useful to study the mechanisms of acquired resistance using these mouse pancreatic cancer models.

Loss of heterozygosity (LOH) is often detected in tumors developing in *BRCA1*/2 mutant carriers, indicating loss of the wild type allele is a critical step in initiation of carcinogenesis [64]. However, we did not observe LOH in heterozygous *Brca1* ^*flox/+*^- or *Brca2* ^*flox/+*^*-KPC* tumors. Among pancreatic tumors of patients with *PALB2*, as for *BRCA1/2* mutation carriers, LOH has been reported [65]. Therefore, using our *Palb2-KPC* mouse model, we tested whether LOH is required for pancreatic tumor development in *Palb2*^*flox/+*^*; KPC* animals. Like *Brca1*^*flox/+*^*; KPC* or *Brca2*^*flox/+*^*; KPC* mice, tumor latency of *Palb2*^*flox/+*^*; KPC* animals was similar to *KPC* animals and the tumors maintained the intact wild type allele as well as functional HR (Fig 5B), which is consistent with previous reports showing that deletion one allele of *Palb2*, *Brca1* or *Brca2* in mice did not affect tumor frequency or genome stability [16, 66].

In summary, we have generated *Palb2*, *Brca1* and *Brca2* deleted mouse models for pancreatic cancer development and compared their tumor phenotypes, as summarized in Fig 7. Improved preclinical models such as our triple mutant pancreatic tumor models, recapitulating the pathogenesis of human pancreatic cancer, combined with powerful high-throughput screening techniques will help to identify novel diagnostic/therapeutic targets for familial pancreatic cancer and overcome mechanisms of drug resistance. Moreover, these animal models will be useful to test new therapeutic regimens, drug combination therapies and immune therapy for both familial and sporadic pancreatic cancer patients having mutations in BRCA pathway genes.

## Supporting information

S1 Table List of PCR genotyping primers. (TIF)

S1 Supporting Information Genotype confirmation of mouse pancreatic tumor cells. (TIF)

S2 Supporting Information Immunofluorescence staining for CK19 on pancreatic tumor cells. (TIF)

S3 Supporting Information Uncropped row images of gels and films. (TIF)
