# Supplementary figures and images for: Murine models for familial pancreatic cancer: Histopathology, latency and drug sensitivity among cancers of Palb2, Brca1 and Brca2 mutant mouse strains
Source: PLoS One. 2019 Dec 26;14(12):e0226714. doi: 10.1371/journal.pone.0226714 (PMC6932818; doi:10.1371/journal.pone.0226714)

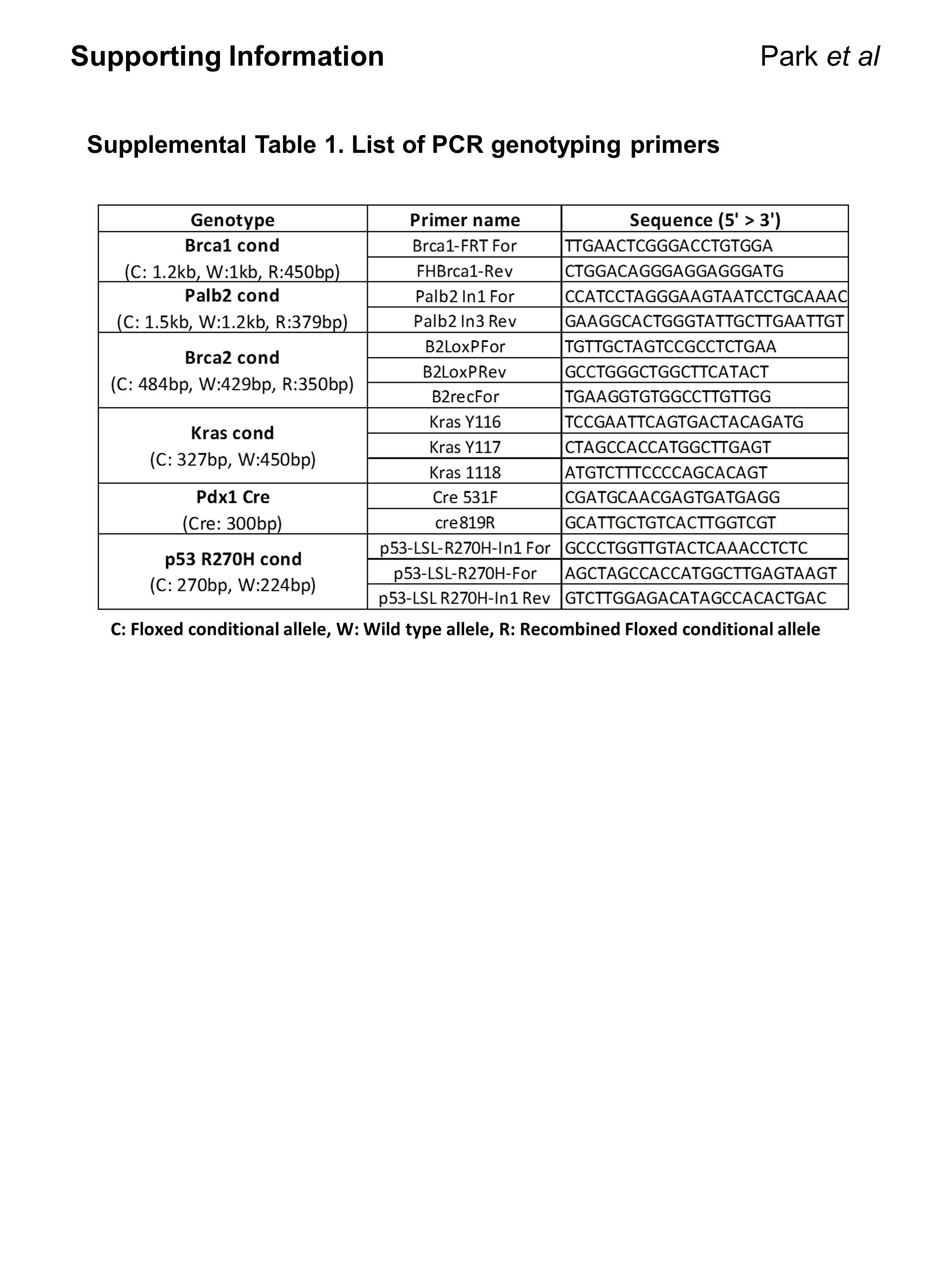

Supplement: S1 Table — (TIF) [file pone.0226714.s001.tif]

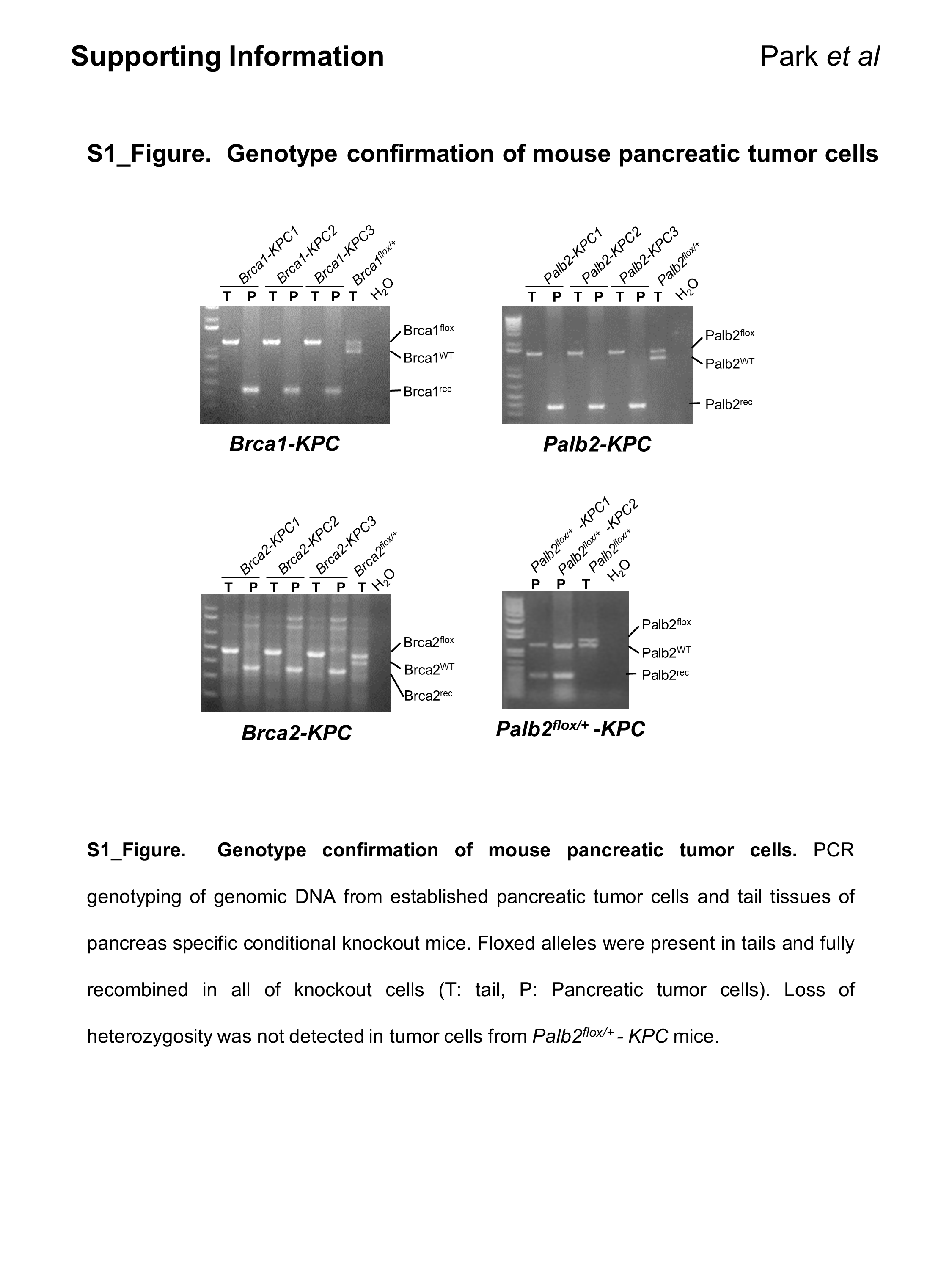

Supplement: S1 Supporting Information — (TIF) [file pone.0226714.s002.tif]

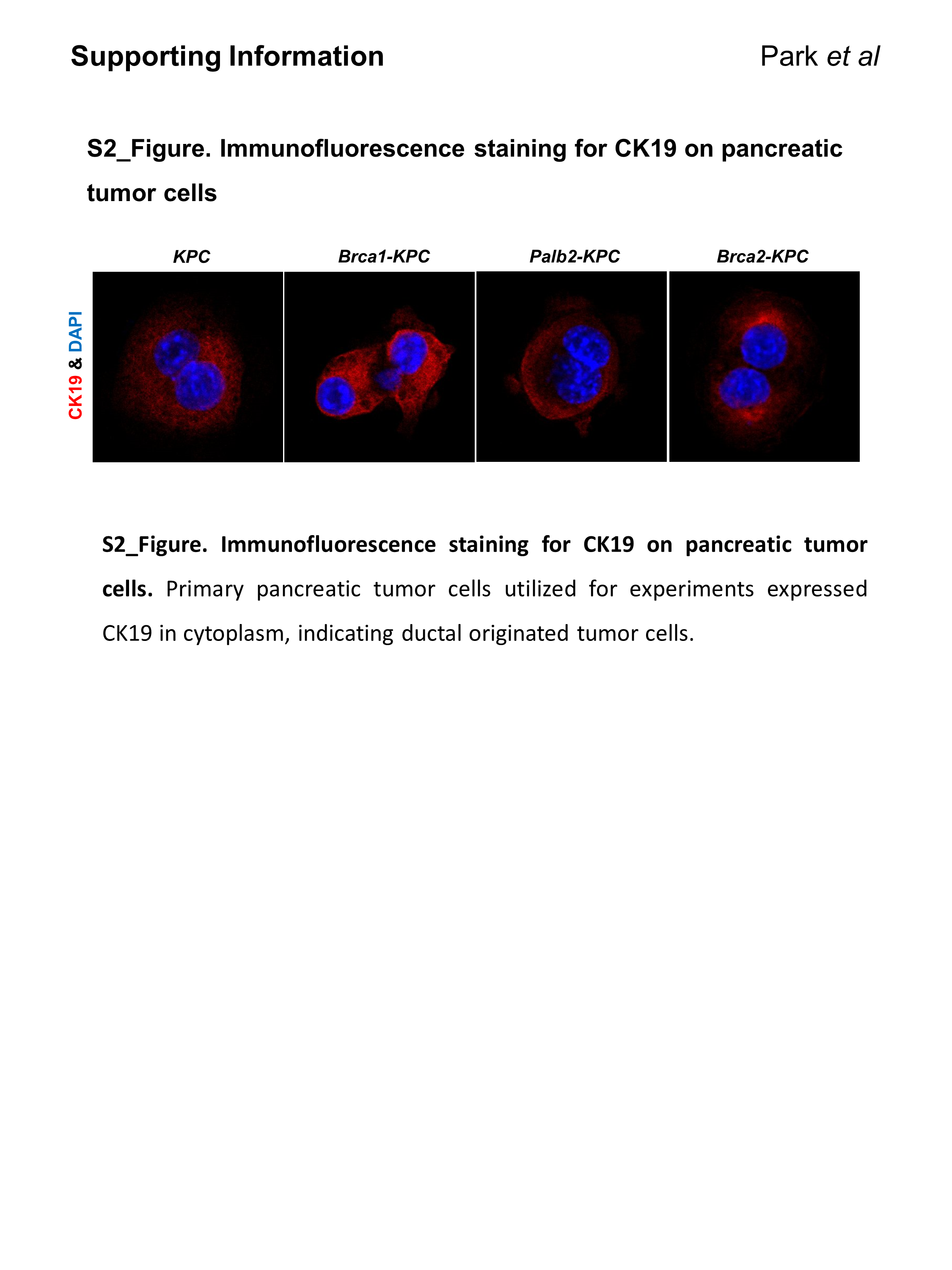

Supplement: S2 Supporting Information — (TIF) [file pone.0226714.s003.tif]

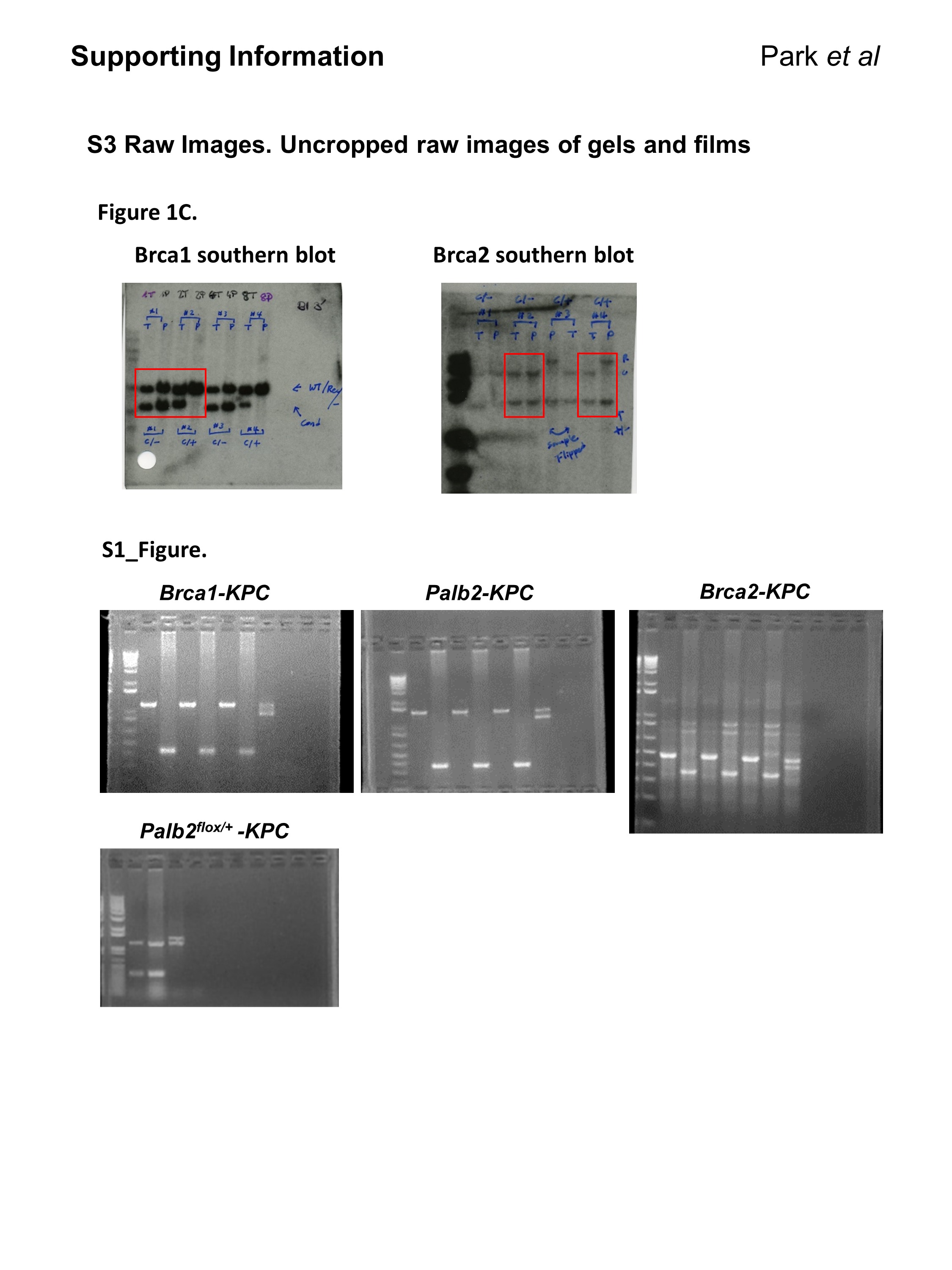

Supplement: S3 Supporting Information — (TIF) [file pone.0226714.s004.tif]
